# Does Quantification of [^11^C]*meta*-hydroxyephedrine and [^13^N]ammonia Kinetics Improve Risk Stratification in Ischemic Cardiomyopathy

**DOI:** 10.1007/s12350-021-02732-5

**Published:** 2021-08-02

**Authors:** Jean Z. Wang, Jason G. E. Zelt, Nicole Kaps, Aaryn Lavallee, Jennifer M. Renaud, Benjamin Rotstein, Rob S.B. Beanlands, James A. Fallavollita, John M. Canty, Robert A. deKemp

**Affiliations:** 1 Department of Medicine (Cardiology), University of Ottawa Heart Institute, Ottawa, Canada; 2 Faculty of Medicine, University of Ottawa, Ottawa, Canada; 3 INVIA Medical Imaging Solutions, Ann Arbor, MI, USA; 4 VA Western New York Healthcare System, Buffalo, NY, USA; 5 Division of Cardiovascular Medicine, University at Buffalo, Buffalo, NY, USA

**Keywords:** Sympathetic Innervation, Heart Failure, Positron Emission Tomography, Myocardial Perfusion

## Abstract

**Background:**

In ischemic cardiomyopathy patients, cardiac sympathetic nervous system dysfunction is a predictor of sudden cardiac arrest (SCA). This study compared abnormal innervation and perfusion measured by [^11^C]*meta*-hydroxyephedrine (HED) vs [^13^N]ammonia (NH_3_), conventional uptake vs parametric tracer analysis, and their SCA risk discrimination.

**Methods:**

This is a sub-study analysis of the prospective PAREPET trial, which followed ischemic cardiomyopathy patients with reduced left ventricular ejection fraction (LVEF≤35%) for events of SCA. Using n=174 paired dynamic HED and NH_3_ positron emission tomography (PET) scans, regional defect scores (%LV extent×severity) were calculated using HED and NH_3_ uptake, as well as HED distribution volume and NH_3_ myocardial blood flow by kinetic modelling.

**Results:**

During 4.1 years follow-up, there were 27 SCA events. HED defects were larger than NH_3_, especially in the lowest tertile of perfusion abnormality (p<0.001). Parametric defects were larger than their respective tracer uptake defects (p<0.001). SCA risk discrimination was not significantly improved with parametric or uptake mismatch (AUC=0.73 or 0.70) compared to HED uptake defect scores (AUC=0.67).

**Conclusion:**

Quantification of HED distribution volume and NH_3_ myocardial blood flow produced larger defects than their respective measures of tracer uptake, but did not lead to improved SCA risk stratification vs. HED uptake alone.

## Background

Cardiac sympathetic nervous system dysfunction contributes to the pathogenesis of various conditions, including myocardial ischemia, arrhythmias and congestive heart failure.[1–3] Quantifying the burden of sympathetic denervation using [^11^C]*meta*-hydroxyephedrine (HED) for cardiac PET imaging is predictive of SCA in patients with ischemic cardiomyopathy, as demonstrated by the Prediction of ARrhythmic Events with Positron Emission Tomography (PAREPET) trial.[4] As a structural analog of norepinephrine, HED shares similar neuronal kinetic properties of norepinephrine with a high affinity for the norepinephrine reuptake transporter (NET-1), but is resistant to degradation and thus undergoes packaging into presynaptic storage vesicles.[3,5,6] HED PET is widely considered the gold standard for the non-invasive assessment of sympathetic innervation and function.[7]

In patients with ischemic cardiomyopathy, the degree of sympathetic denervation is often equivalent or larger than the perfusion defect.[8] Therefore, sympathetic neurons are thought to be more sensitive to ischemic damage than cardiomyocytes.[9] These zones of perfusion-denervation mismatch are hypothesized to be important substrates for the development of lethal arrhythmias.[10,11] Imaging of cardiac sympathetic denervation has been integrated with myocardial perfusion data to investigate these mismatch zones in preclinical studies but has yet to be rigorously evaluated in human populations.[12,13] It is also unknown to what degree HED yields prognostic information independent of blood flow, as tissue perfusion to the myocardium is required for tracer delivery and uptake. As a result, it currently remains under debate whether HED is simply a pseudo-marker of perfusion.

Various tracer quantification methods, using graphical or kinetic models, have been proposed to quantify HED uptake over time.[14–16] To date, the assessment of HED uptake in clinical trials has been primarily restricted to semi-quantitative measures such as tracer uptake and retention index. This may have important clinical implications, particularly when quantifying regional defects, as these measures can be prone to inaccurate results which are influenced by cardiac motion, partial volume effects such as blood and tissue spill-over, and the contribution of intravascular activity.[7] Tracer kinetic modelling for measurement of the volume of distribution can be employed to overcome these shortcomings but remains experimental. Few studies have fully characterized tracer kinetic parameters obtained through parametric analysis compared with conventional tracer uptake results. It is currently unknown whether full tracer dynamic analysis of HED provides additional clinical information compared to static uptake images alone, as well as its role in SCA risk discrimination.

The primary objectives of this study were threefold, 1) to compare the extent and severity of regional abnormalities in left ventricular (LV) myocardial perfusion vs sympathetic innervation; 2) to compare conventional uptake vs parametric measures of perfusion abnormality and sympathetic denervation; and 3) to compare the SCA risk discrimination ability of these parameters.

## Methods

### Patient Population and Study Design

This is a sub-study analysis of dynamic HED and NH_3_ PET imaging data from the PAREPET trial (NCT01400334).[4] The aim of the original PAREPET trial was to investigate whether imaging of hibernating myocardium and sympathetic dysfunction predicts SCA. The prospective observational trial included 204 participants with documented ischemic cardiomyopathy, an ejection fraction (LVEF) of ≤ 35%, and no plans for coronary revascularization. Exclusion criteria were previously resuscitated SCA, sustained ventricular tachycardia, implantable cardioverter-defibrillator (ICD) discharge, unexplained syncope, recent myocardial infarction in the last 30 days, percutaneous coronary intervention in the last 3 months, coronary bypass surgery in the last year, and comorbidities leading to a reduced life expectancy < 2 years. The study protocol was approved by the University at Buffalo Institutional Review Board and all subjects signed an informed consent form. Of the 204 enrolled participants, 188 underwent both HED and NH_3_ PET scans.

### Scanning Protocol

Study participants underwent dynamic cardiac PET to quantify resting perfusion using [^13^N]ammonia and sympathetic innervation using [^11^C]*meta*-hydroxyephedrine. The ECAT EXACT HR+ (CTI, Knoxville, Tennessee) PET scanner (15.5 cm axial field-of-view; resolution ~5.4 mm^3^ full-width-at-half-maximum) was used to perform PET imaging for this study, with 3 rotating ^68^Ge rod sources used to perform attenuation correction. Sympathetic innervation was assessed with 740 MBq of HED, and perfusion was assessed with 740 MBq of NH_3_. HED and NH_3_ were administered by bolus injection and dynamic imaging was captured for HED (60 minutes) and NH_3_ (19 minutes). The following frame sequence was used for HED dynamic imaging: 6 x 30 s, 2 x 60 s, 2 x 150 s, 2 x 300 s, 2 x 600 s, 1 x 1200 s, and for NH_3_ dynamic imaging: 12×5 s, 4 × 15 s, 2 × 30 s, 2 × 60 s, 1 × 240 s, and 2 × 300 s. The HED images were decay corrected and reconstructed using filtered back-projection and 12 mm Gaussian post-filtering, with the final images at 13 mm full-width-at-half-maximum resolution.

### PET Image Analysis

**Fig. 1:**
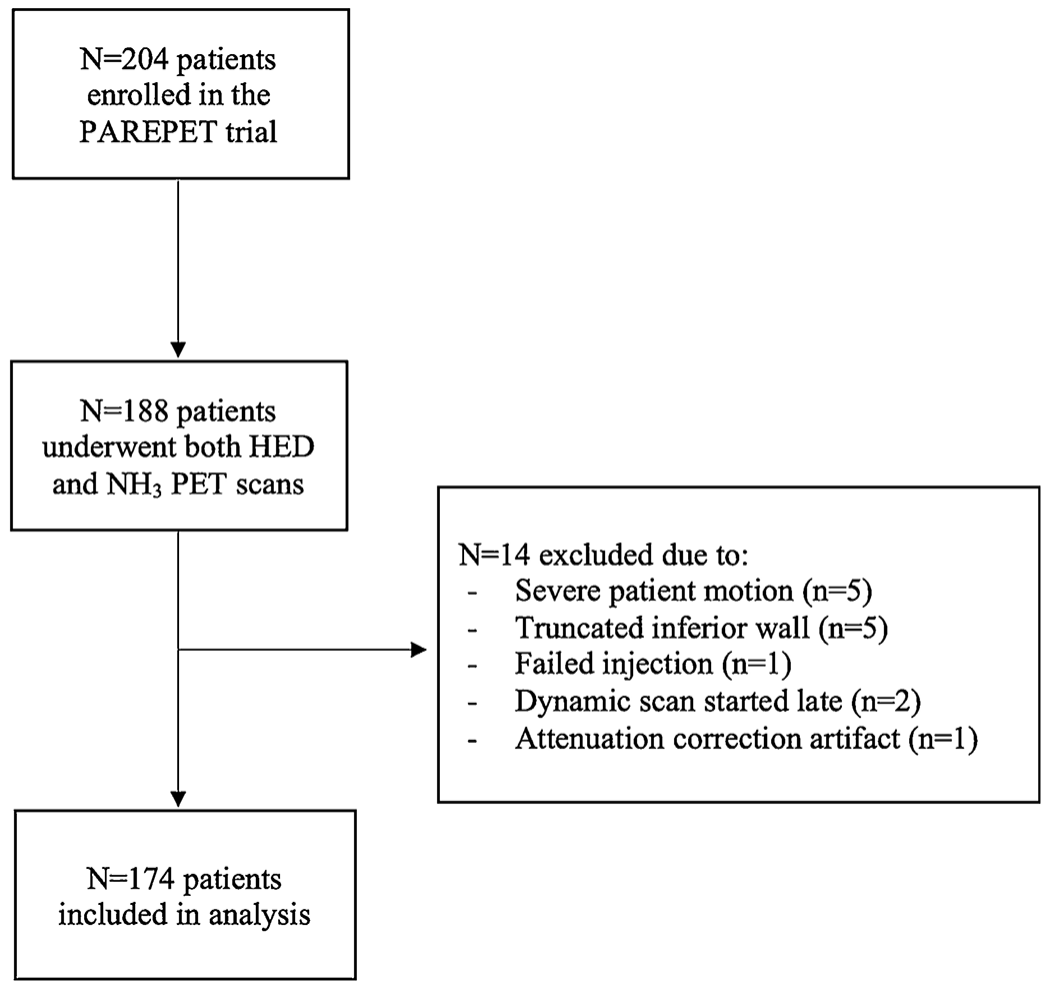
Patient Flow Diagram and Reasons for Exclusion

The quality of all scans was reviewed, based on factors such as image contrast and noise, as well as adequate spatial and temporal sampling. Supplementary Figure 1 illustrates a comparison of polar maps between a poor-quality excluded scan compared to an adequate quality included scan. N=174 scan pairs were identified for analysis, with n=14 scans excluded from the present analysis due to reasons listed in Figure 1. The scans were analyzed using in-house developed software, FlowQuant® v2.5 (University of Ottawa Heart Institute, Ottawa, ON, Canada). One-tissue compartment modeling, regional denervation score, and NH_3_-HED mismatch analysis techniques were incorporated into the software as follows.

### Tracer Kinetic Modelling

#### HED Volume of Distribution

The distribution volume (DV) is defined as the ratio of the tracer concentration in the myocardium (*C_m_*) to the tracer concentration in arterial plasma (*C_p_*) at equilibrium (T_E_).[17]


(1) $$DV=\frac{C_{m}\left(T_{E}\right)}{C_{p}\left(T_{E}\right)},\;when\;\frac{dC_{m}}{dt}=0$$

The DV (mL/g) represents the tracer-transporter binding affinity (i.e. B_max_/K_d_), where B_max_ is the NET-1 transporter density and 1/K_d_ is the affinity of the tracer (HED) for the transporter.[17] While tracer concentrations would require a long time to reach equilibrium, the DV can be computed using a one-compartment model through dynamic imaging. A simple one-tissue compartment model (1TCM) was used to analyze the full kinetics of HED (and 0-4 min for NH_3_) as it has been shown to provide more robust estimates of DV with optimal clinical reproducibility compared to the two-tissue compartment model.[7] Using a reversible 1TCM, the rate-of-change of tracer concentration in the myocardium is defined by the rate of tracer influx from arterial plasma-to-myocardium (K_1_) and the efflux from the myocardium (k_2_) through the following equation:[15]


(2) $$\frac{dC_{m}}{dt}=K_{1}C_{p}\left(t\right)-k_{2}C_{m}\left(t\right)$$

At equilibrium, the rate of change of tracer concentration in the myocardium is equal to zero ($\frac{dC_{m}}{dt}=0$). Thus, the distribution volume can be expressed as the following equation by combining equation 1 and 2:

(3) $$DV=\frac{K_{1}}{k_{2}}=\frac{C_{m}\left(T_{E}\right)}{C_{p}\left(T_{E}\right)}$$

#### Blood Metabolite Corrections

The arterial whole blood time-activity curve C_WB_(t) was obtained from a 20 mm^3^ region-of-interest positioned automatically in the LV cavity. Radiolabelled metabolites that accumulate in the blood plasma, not present in the myocardium, were corrected to ensure accuracy of the quantitative HED and NH_3_ analyses.[18] The following equation was used to correct for blood metabolites, where the unchanged parent tracer concentration in plasma, C_p_(t), is related to the arterial whole-blood tracer concentration, C_WB_(t) using the parent fraction in plasma function, pfp(t) as determined previously in human subjects for HED and NH_3_:[7,19]


$$C_{p}\left(t\right)=C_{WB}\left(t\right)\times pfp\left(t\right)$$

#### Partial Volume Correction

Blurring of the acquired PET images in the LV myocardium *C_LV_(t)* due to contractile and respiratory motion leads to underestimation of myocardial tissue concentrations, and there is also a fraction of blood volume (FBV) within normal myocardial tissue. As such, partial volume correction was utilized as part of the 1TCM to correct for these effects.[20,21] The geometric partial volume correction model utilized in this study, as well as others, is defined by the following equation:[15]


(4) $$C_{LV}\left(t\right)=\left(1-FBV\right)\times C_{m}\left(t\right)+FBV\times C_{WB}\left(t\right)$$

#### NH_3_ Myocardial Blood Flow

The delivery rate constant, K_1_, is equal to the product of blood flow and unidirectional extraction fraction (*E* ’) during first pass of the tracer. The following equation was utilized to measure myocardial blood flow (MBF) with NH_3_ through its standard relationship with the permeability surface area product (PS) and extraction fraction:[22]


(5) $$E^{\prime }=MBF\times \left(1-e^{-PS/MBF}\right)$$

#### Regional Defects

**Fig. 2:**
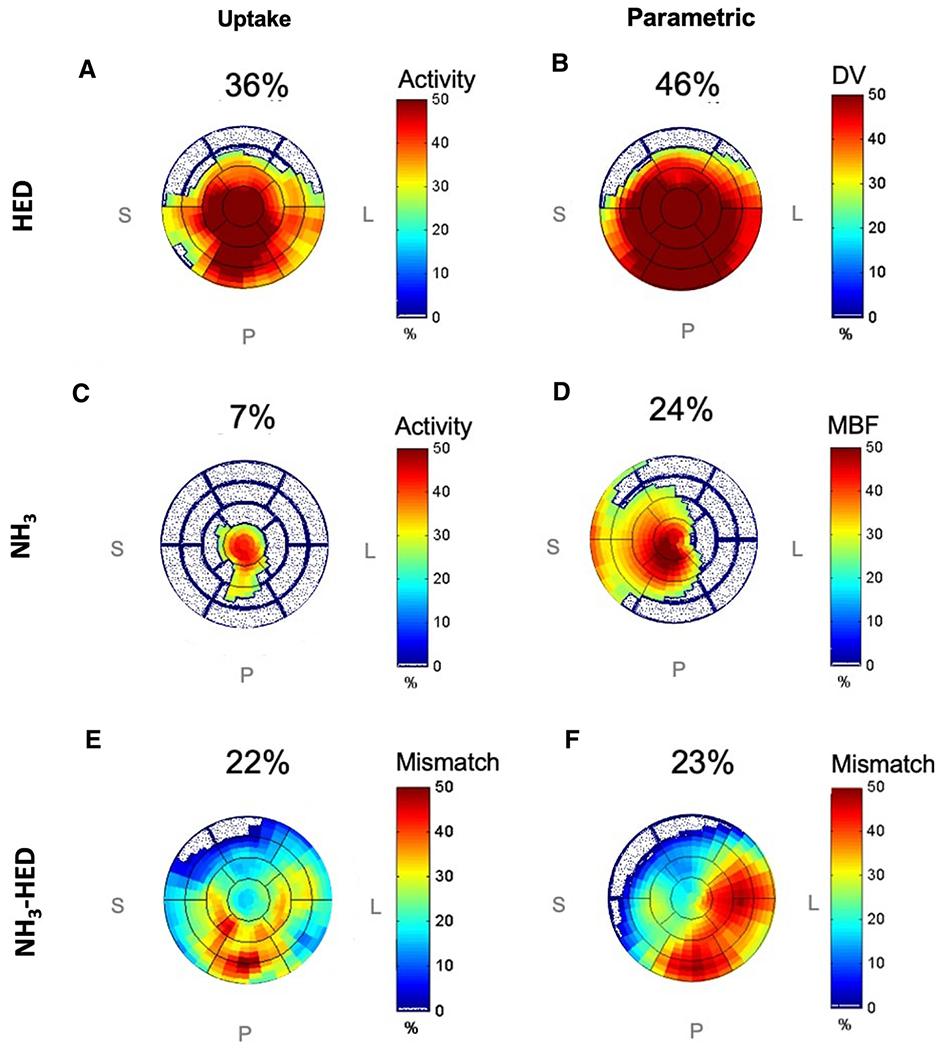
Left ventricular (LV) defect polar maps for HED (A-B), NH_3_ (C-D) and NH_3_-HED mismatch for a patient with an apical defect. Normal regions are shown in white (defect = 0%). The maps demonstrate similar location of defects, but the parametric DV and MBF values obtained via compartment modelling (B, D) show larger defect scores than the corresponding uptake values (A, C). The HED defects (A, B) also appear visually larger than the NH_3_ defects (C, D). Defect scores listed above each polar map are expressed as %LV extent × severity.

HED and NH_3_ uptake, parametric, and mismatch measures were all expressed as regional defect scores which reflect the extent × severity of relative defects, as we have previously established in the literature.[23] Steady-state tracer uptake was measured by averaging dynamic HED images from 15 to 60 minutes and NH_3_ images from 3 to 19 minutes post-injection. The LV myocardium activity was sampled automatically into 496 polar-map sectors (P) and normalized (0-100%) relative to the peak activity. The defect scores were then calculated as the extent × severity of polar-map sectors <75% of the maximum and expressed as a percentage of the left ventricle (%LV). Figure 2 illustrates an example of HED and NH_3_ regional denervation, perfusion, and mismatch polar-map scores.

Perfusion-innervation mismatch was calculated as %LV by averaging positive values of the NH_3_ minus HED polar-map using the following equation:

$$\mathrm{Mismatch score}=\frac{\sum_{i=1}^{P}\left(NH3_{i}-HED_{i}\right)>0}{P}$$

A threshold of 75% was chosen based upon the methodology of the original PAREPET trial, which is consistent with previous PET myocardial perfusion imaging methodology reported in the literature.[24,25] Differences in positron range between N-13 and C-11 (or future F-18-labeled tracers) should have negligible effect on defect scores derived from the PET images reconstructed with 12 mm post-filtering.

### Statistical Analysis

The comparisons of innervation vs perfusion as well as uptake vs parametric defect scores were conducted with linear regression, Bland-Altman analysis, and Wilcoxon Signed-Rank tests. The limits-of-agreement of repeated measures was calculated as the mean difference ± coefficient-of-repeatability (RPC=1.96×SD). To evaluate flow-dependence of HED uptake and clearance, the %LV defect scores were analyzed within tertiles of NH_3_ uptake scores. Time-to-SCA was analyzed using the Kaplan-Meier method, and differences between tertiles were assessed by the Log-Rank test. A cox-proportional hazards regression was also used to assess the association between the HED and NH_3_ PET parameters and SCA. Area under the receiver operator characteristic curve (AUC) was used to assess the ability of the individual HED and NH_3_ PET parameters to discriminate between patients who developed SCA vs. those who did not. Statistical analysis and graphical representation were performed using SAS 9.4 (SAS Institute, NC), GraphPad Prism 6.0 (GraphPad, La Jolla, CA, USA), SPSS (IBM), and Excel 2018 (Microsoft). For all analyses, statistical significance was defined as a p-value < 0.05.

## Results

### Patient Demographics

The patient demographics are listed in Table 1. The average age of the population was 67±12 years with 90% males. Most of the patients were NYHA class II and CCS Angina class II, with an average LVEF of 28±9%. During an average follow up of 4.1 years, there were 27 events of sudden cardiac arrest.

### Comparison of HED vs NH_3_ PET Imaging

**Fig. 3:**
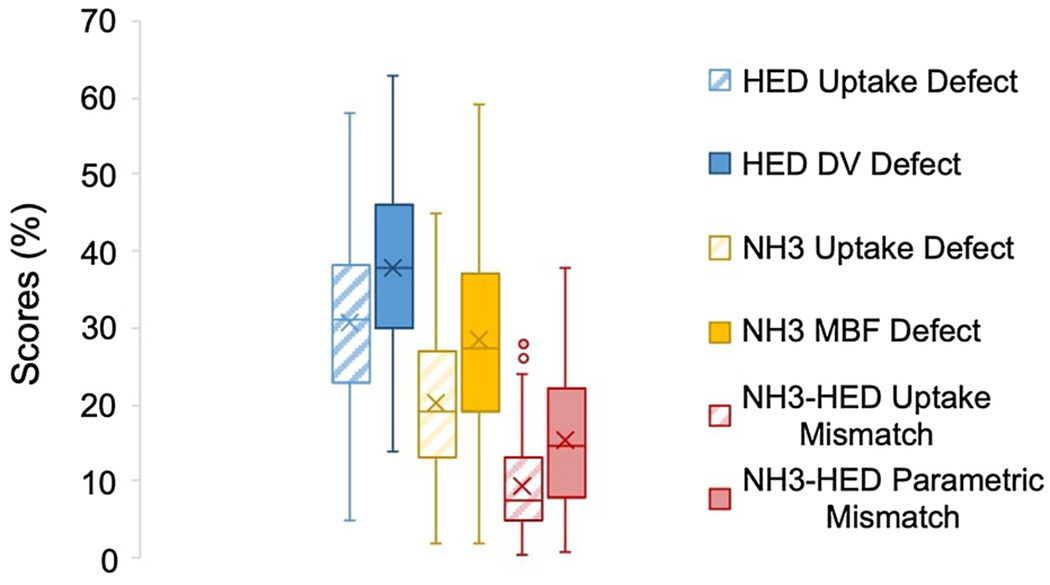
Box plot of myocardial perfusion, sympathetic denervation and mismatch scores, demonstrating that parametric scores are larger than the corresponding uptake scores.

**Fig. 4:**
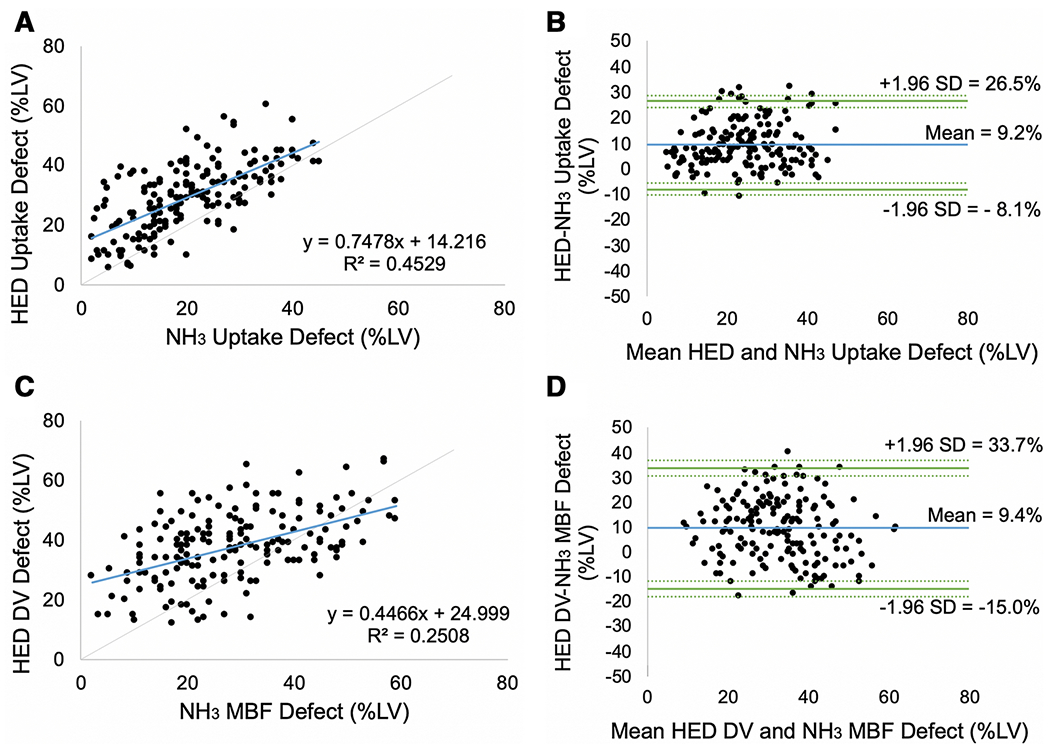
Correlation (A, C) and Bland Altman (B, D) diagrams comparing HED vs NH_3_ defect scores (%LV) using tracer uptake (A, B) and parametric measures of HED distribution volume vs NH_3_ myocardial blood flow (C, D).

Sympathetic denervation measured by HED PET imaging detected larger defects on average than myocardial perfusion imaging with NH_3_, as depicted in Figure 3 and Figure 4. When comparing tracer uptake values, the average LV defect extent and severity measured by the HED defect score was 9.2±8.8% larger than the NH_3_ defect score (p<0.001). A similar trend was noted for the parametric measures. The HED LV distribution volume defect score was 9.4±12.4% larger than the NH_3_ myocardial blood flow defect score (p<0.001).

### Flow Dependence of HED

**Fig. 5:**
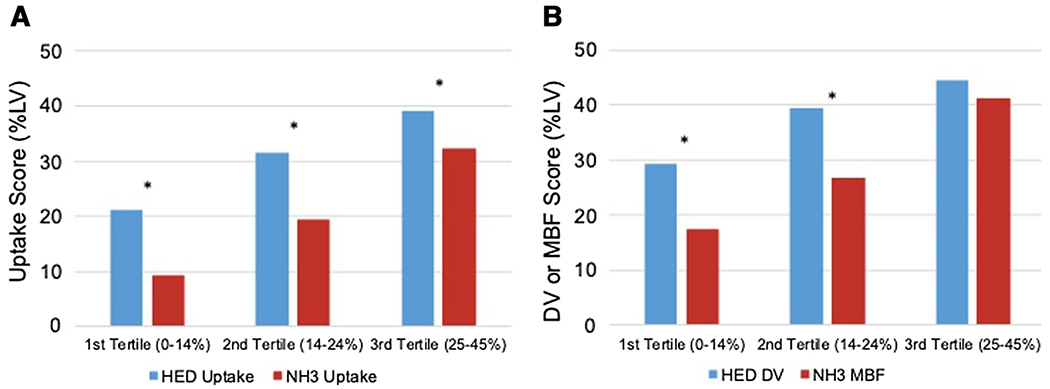
Comparison of HED and NH_3_ defect scores by tertiles of perfusion defect, for HED and NH_3_ uptake scores (A) and parametric values of HED DV and NH_3_ MBF (B). The largest differences between HED and NH_3_ defect scores are observed in patients with the smallest perfusion defects, i.e. 1^st^ tertile. *P<0.05 between tracers.

To assess the flow dependence of HED, the results were grouped into tertiles of NH_3_ uptake defect scores. The average HED scores were compared with NH_3_ scores in each tertile (see Figure 5). The largest differences between HED and NH_3_ scores were noted in the lowest tertile of NH_3_ uptake defects; the HED defect score was on average 11.8±9.8% larger than the NH_3_ defect scores (p<0.001). However, in the highest tertile of perfusion defects, the HED defect score was only 6.8±8.7% larger than the NH_3_ defect scores (p<0.001).

A similar trend was seen in the parametric comparison of HED distribution volume defect scores vs NH_3_ myocardial blood flow scores. In the lowest tertile of perfusion defects, the difference between the average defect size measured by the HED distribution volume scores and NH_3_ myocardial blood flow scores was 11.9±12.9% (p<0.001) compared to a non-significant difference of 3.4±9.2% (p=0.08) in the highest tertile of perfusion defects.

### Comparison of Parametric and Uptake Scores

**Fig. 6:**
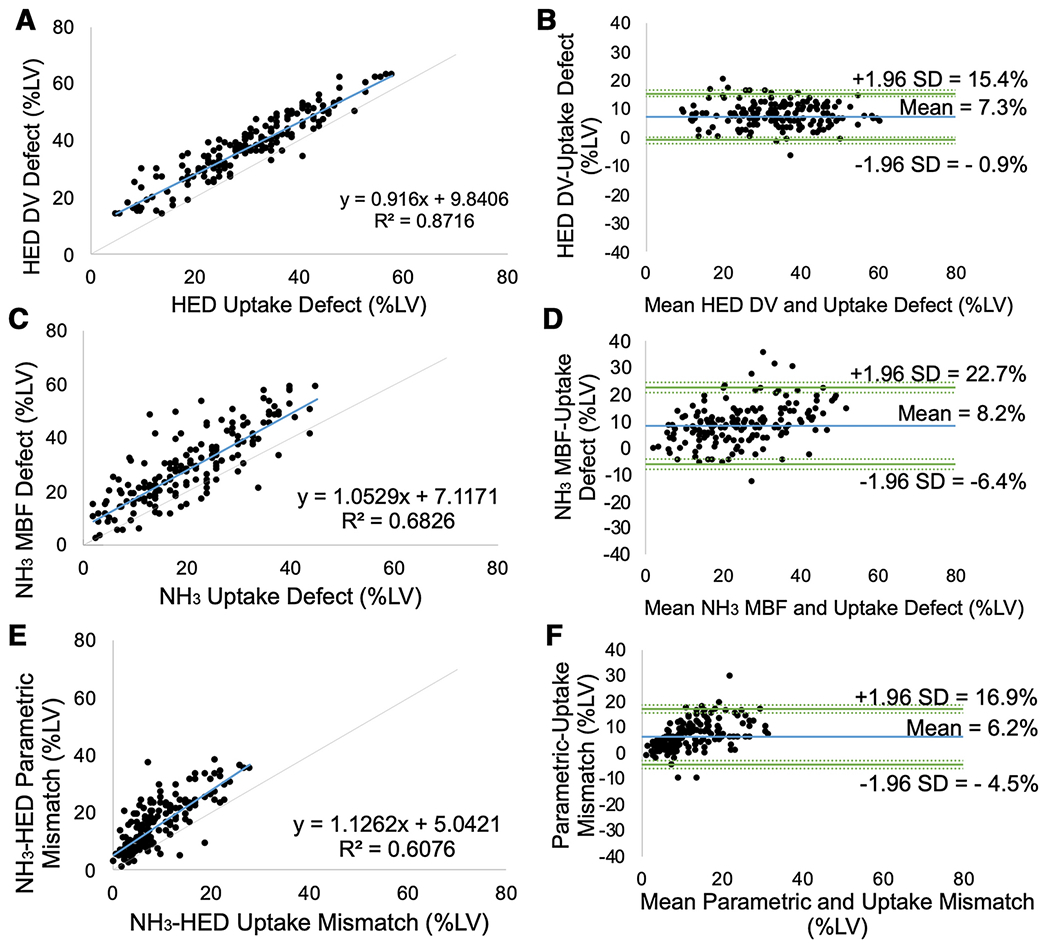
Comparison of uptake vs parametric defect scores for HED, NH_3_, and mismatch. Correlation (A, C, E) and Bland Altman diagrams (B, D, F) for HED uptake compared to DV defects (A-B), NH_3_ uptake vs myocardial blood flow defects (C-D), and parametric vs uptake NH_3_-HED mismatch scores (E,F).

Defects assessed using parametric measures of tracer kinetics (i.e HED distribution volume and NH_3_ myocardial blood flow scores) were larger than their respective uptake scores, as shown in Figure 6. The average sympathetic innervation defect size measured by the HED distribution volume score was 7.3±4.2% larger than the uptake score (p<0.001). The average defect size measured by the NH_3_ myocardial blood flow score was 8.2±7.4% of the LV greater than the NH_3_ uptake score (p<0.001). Parametric NH_3_–HED mismatch scores were significantly larger than their respective uptake mismatch scores (Δ=6.2±5.5%, p<0.001).

### Prediction of SCA with HED and NH_3_ Parameters

**Fig. 7:**
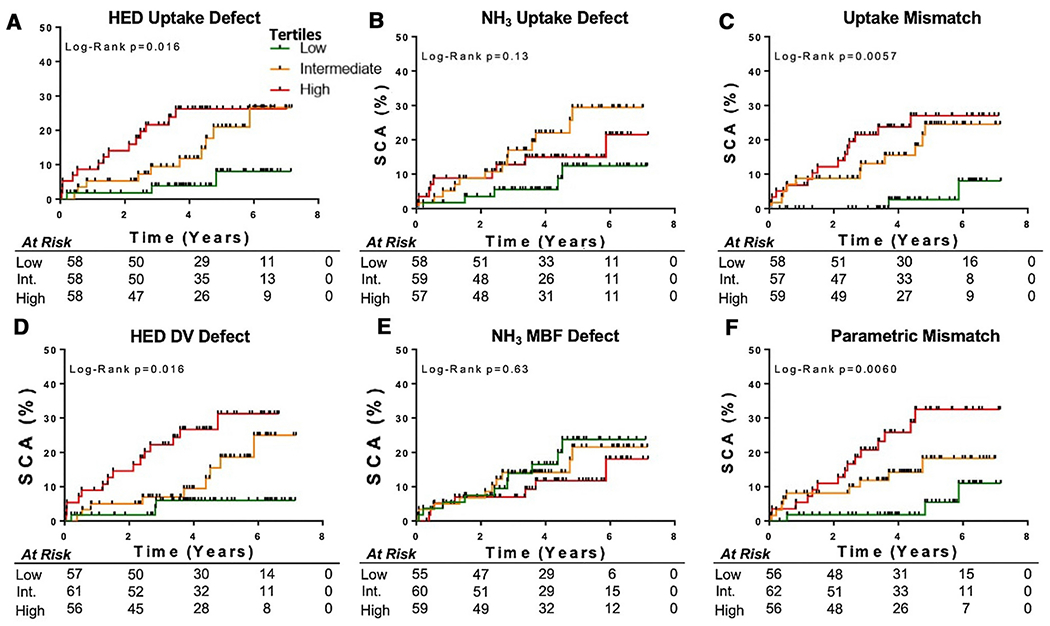
Kaplan Meier curves demonstrating incidence of SCA in tertiles of HED and NH_3_ innervation and perfusion parameters. HED uptake defect (A), HED DV defect (D), NH_3_-HED uptake mismatch (C), and MBF-DV parametric mismatch (F) scores were significant predictors of SCA (P<0.05), whereas NH_3_ uptake defect (B) and NH_3_ MBF defect (E) scores were not (P>0.05).

**Fig. 8:**
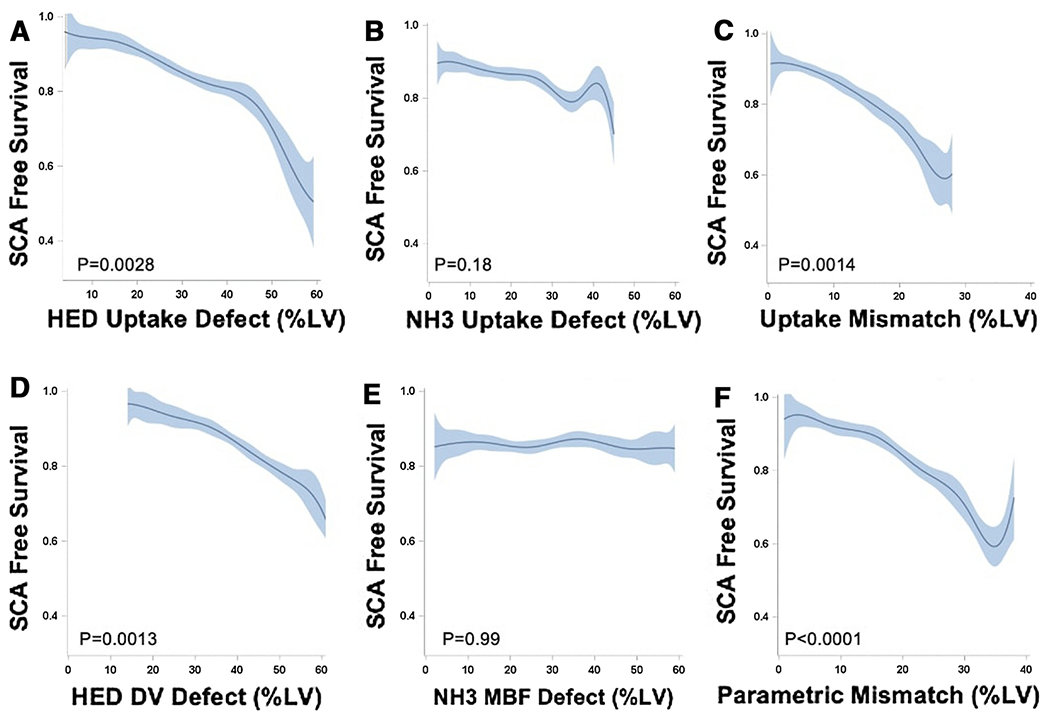
SCA-free survival as continuous functions of PET HED and NH_3_ defect scores, and NH_3_-HED mismatch scores. Cox regression analysis revealed that denervation scores (A, D) and NH_3_-HED mismatch scores (C,F) were associated with SCA (P<0.05), but not NH_3_ or MBF defect scores alone (B,E).

The Kaplan Meier curves in Figure 7 demonstrate that uptake and parametric scores of HED and perfusion-innervation mismatch were predictive of sudden cardiac arrest (P=0.006-0.016), whereas NH_3_ uptake and parametric scores were not (P>0.05). This is also reflected in the continuous survival curves shown in Figure 8, where the SCA-free survival decreased with increasing HED and mismatch parameters, whereas NH_3_ uptake and parametric (MBF) scores were not statistically significant predictors. These survival curves have fluctuations in the NH_3_ uptake and parametric mismatch curves near the extremes of defect sizes, likely due to a small sample size of patients with very large defects.

### SCA Risk Discrimination

**Fig. 9:**
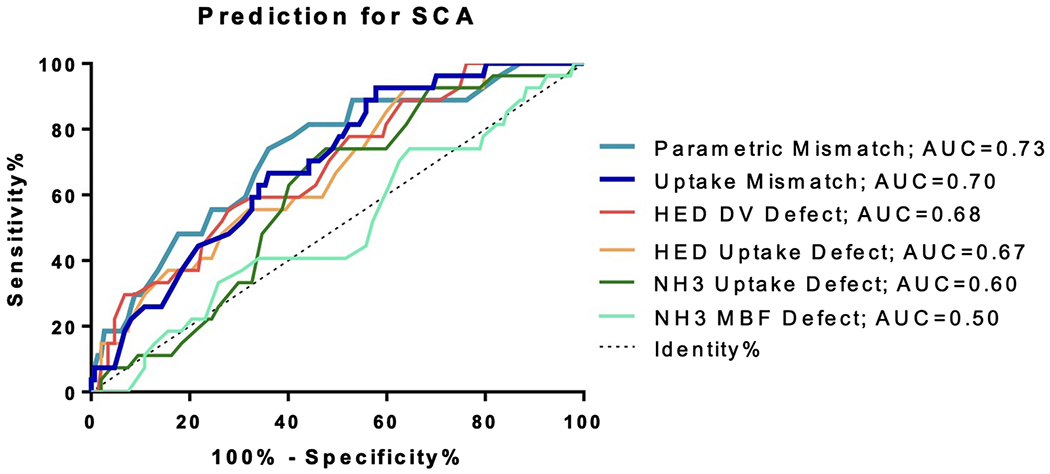
Comparison of SCA risk discrimination for various HED and NH_3_ parameters, with AUC values listed in the legend.

For each NH_3_ and HED parameter, the receiver operator characteristic (ROC) curves and AUC values for SCA risk discrimination are found in Figure 9 and Table 2. The parametric mismatch score had the highest AUC for prediction of SCA (AUC=0.73), however this was not significantly higher than the HED uptake score alone (ΔAUC=0.059, 95%CI [−0.059 to 0.175], p=0.33) as shown in Table 2. Even addition of the parametric mismatch score to the HED uptake defect score in a combined assessment did not significantly improve the AUC over the HED defect score alone (ΔAUC=−0.073, 95%CI [−0.160 to 0.014], p=0.10). HED uptake, HED DV, NH_3_-HED uptake mismatch and NH_3_-HED parametric mismatch were strong predictors of SCA using the cox-proportional hazards model (P=0.001-0.003), however NH_3_ parameters were not predictive (P=0.174-0.994), as shown in Table 3.

## Discussion

This present study represents the first comparison of parametric and uptake score measures of cardiac sympathetic denervation and perfusion using HED and NH_3_ PET tracers, respectively. A head-to-head comparison of HED sympathetic denervation with NH_3_ myocardial blood flow measurements was also a novel aspect of this study, which we anticipate will have important clinical implications. Four key findings were derived from this study in patients with ischemic cardiomyopathy: (1) HED scores, both uptake and parametric, detected larger defects than their respective NH_3_ scores. This provides additional data supporting the paradigm of sympathetic neurons being exquisitely sensitive to ischemia, possibly more so than cardiomyocytes. (2) The largest differences between HED and NH_3_ defect size occurred in the lowest tertile of perfusion defects where resting perfusion was relatively preserved. This suggests that denervation may occur in the absence of myocardial scar. (3) The parametric measures of HED distribution volume, NH_3_ myocardial blood flow, and NH_3_-HED mismatch detected significantly larger defects than their respective counterparts, HED uptake, NH_3_ uptake, and NH_3_-HED uptake mismatch defect. Nevertheless, parametric defects did not significantly improve SCA risk discrimination compared to uptake defect scores. (4) NH_3_ uptake and parametric MBF scores were both poor predictors of SCA. This corroborates previous findings of the PAREPET study suggesting that HED is the strongest predictor of SCA. Taken together, these data provide credence to HED cardiac PET yielding biological information on sympathetic integrity, and independent of myocardial blood flow.

### Comparison of HED vs NH_3_

Defects measured by HED PET imaging were larger than those detected using myocardial perfusion imaging with NH_3_ PET. The averages scores differed by approximately 9-10%LV (p<0.001) when comparing parametric vs. uptake measures. NH^3^ MBF vs uptake defects appeared to have greater variability as shown in Figure 6 compared to HED DV vs uptake defects, likely secondary to increased noise in the NH_3_ images that was further amplified by the MBF kinetic modeling. These findings are consistent with previous studies demonstrating C-1 1-hydroxyephedrine abnormalities are often more extensive than blood flow defects assessed by uptake polar map analysis.[8,26] They support the hypothesis that physiologically, cardiac sympathetic neurons are more susceptible to ischemic damage than cardiomyocytes.[9,27]

Furthermore, HED parameters demonstrated improved SCA risk discrimination compared to NH^3^ parameters. SCA risk discrimination was poor with ammonia, as Kaplan Meier and Cox Proportional Hazards analysis demonstrated that tertiles of NH_3_ uptake and MBF were not predictive of SCA. The AUC values were low for both resting NH_3_ uptake (AUC=0.60) and NH_3_ MBF (AUC=0.50). This supports previous conclusions that resting NH_3_ is a poor predictor of SCA risk in comparison to HED.[4,28] While it has been hypothesized in preclinical studies that NH_3_-HED mismatch zones could be important substrates for developing arrythmias, this present analysis did not demonstrate improved risk stratification with mismatch compared to sympathetic denervation alone.[12,13]

### Comparison of Parametric vs Uptake Defect Scores

HED DV, NH_3_ MBF, and NH_3_-HED mismatch scores detected larger defects than their respective uptake scores by approximately 6-8%LV (p<0.001). Nevertheless, this did not impact the prognostic analysis, as SCA risk discrimination was not significantly improved by parametric vs. simple uptake analysis. Multivariate and interaction analyses were explored, shown in Supplementary Table 1 and Supplementary Figure 2, but the results did not demonstrate any improvement over HED alone. It is possible that this study was underpowered to detect a significant difference, and tracer quantification using automated analysis methods may provide additional information compared to uptake measures with a larger sample size. In the previous literature, a recent analysis of n=254 ischemic heart failure patients demonstrated improved risk stratification with quantitative PET metrics of stress myocardial blood flow, but noted the myocardial flow reserve was only modestly superior to other measures.[29]

Although absolute quantification methods yielded larger defect sizes, they ultimately did not improve SCA risk stratification and there are some limitations to this method of analysis to consider. Quantification techniques such as HED distribution volumes and NH_3_ myocardial blood flow require full tracer dynamic analysis, thus necessitating longer PET imaging times compared to simple static uptake. This analysis also requires high scan quality for appropriate model fit and analysis, as reflected by the N=14 patients excluded from analysis for quality assurance purposes. Furthermore, HED defect scores have been established as a highly reliable and reproducible measure, while previous literature demonstrates slightly lower inter-rater reliability with HED parametric analysis compared to simpler uptake defects.[14,23] The difference in inter-rater variability was small, however, as all HED measures reported to have an ICC>93%.[14]

### Flow Dependency of HED

It has been previously hypothesized that HED is a relatively flow-limited tracer due to its rapid extraction from the blood by NET transporters, resulting in tracer uptake that is more dependent on blood flow rather than neuronal uptake.[16,30] HED requires blood flow to be delivered to target tissues and its effect independent of blood flow has not been conclusively elucidated. This study demonstrates that the largest differences in HED and NH_3_ defect size occur when the perfusion defects are the smallest, i.e. in the lowest tertile of perfusion uptake defects. This finding was consistent for both the parametric and uptake measure comparisons. Taken together, these observations argue against a significant impact of flow limited delivery on HED defect size.

### Limitations

Limitations of this study include the retrospective nature of the analysis, limited sample size, and under-representation of female patients in this study. These factors preclude the comparative assessment of sympathetic imaging markers against other clinical parameters which are associated with SCA. There were also N=14 HED studies excluded from analysis. Due to these excluded studies, the number of SCA events was reduced in this study and thus multivariable modelling was not performed. Reasons that rendered the scans non-diagnostic include a severe motion artifact (n=5), truncated inferior wall (n=5), low-count statistics (n=l), and late scan start-time (n=2). Incomplete measurement of the LV heart volume due to a truncated inferior wall is a common limitation of older PET scanners without x-ray CT available to guide patient positioning. Currently available PET/CT scanners have a larger axial field-of-view and also facilitate attenuation correction and patient positioning to overcome these limitations in contemporary clinical practice. It should be noted that the cardiac perfusion and sympathetic imaging techniques are currently limited to centres with an on-site cyclotron and expertise in cardiac PET innervation imaging analysis.

### New Knowledge Gained

This study provides novel insights into cardiac physiology which supports further study of SNS PET imaging in risk stratifying patients who are the most likely to benefit from a primary prevention ICD to prevent SCA. While parametric analysis of HED, NH_3_, and NH_3_-HED mismatch detects larger defect sizes, our analyses demonstrated that there was no improved SCA risk stratification associated with these measures compared to simple uptake analysis. This simplifies the imaging protocol and analysis methods required for SCA discrimination in future clinical applications, as extensive tracer quantification measures may not be required to provide SCA risk prediction which is adequately captured in simple tracer uptake analysis. Furthermore, radiation ablation of VT is a growing field which utilizes non-invasive myocardial imaging to identify target substrates.[31] There may be potential future applications of sympathetic imaging to identify scar border zone and viable myocardial for VT ablation, however, this has not been well explored in the current literature.

## Conclusion

Cardiac innervation defects measured by HED PET imaging are larger on average than perfusion defects measured by NH_3_ PET imaging. The largest differences between HED and NH_3_ defect sizes occurred when perfusion defects were low, suggesting higher sensitivity to detect denervation early during the course of ischemic heart disease progression. Parametric and uptake defects of HED and NH_3_-HED mismatch were associated with SCA in the univariate analysis, while NH_3_ alone was not predictive of SCA. Quantification of HED and NH_3_ cardiac PET imaging markers detected numerically larger defects but there was no apparent prognostic advantage from parametric imaging compared to conventional uptake defect scores.

## Supplementary Material

1752083_Sup_tab-1

1752083_Sup_file-1

1752083_Sup_file-2

1752083_Sup_Vdo-1

## Figures and Tables

**Table 1.** Baseline Demographic Characteristics

Variable	Value (n=174)
**Demographics**	
Age (years)	67±12
Body mass index (kg/m^2^)	29±5
Male	157 (90%)
Left ventricular ejection fraction (%)	28±9
Diabetes Mellitus	81 (47%)
History of Revascularization	135 (78%)
CCS Angina Class	
Class I	65 (37%)
Class II	82 (47%)
Class III	26 (15%)
Class IV	1 (1%)
NYHA Class	
Class I	33 (19%)
Class II	89 (51%)
Class III	40 (23%)
Class IV	12 (7%)
**Medications**	
Beta-blocker	167 (96%)
Amiodarone	17 (10%)
Antiplatelet therapy (ASA or Clopidogrel)	152 (87%)
Warfarin	71 (41%)
Angiotensin inhibition therapy (ACE inhibitor or ARB)	157 (90%)
Aldosterone antagonist	67 (39%)
Digoxin	67 (39%)
**Laboratory Variables**	
Creatinine (mg/dL)	1.4±0.9
B-type Natriuretic Peptide (pg/L)	421±454
Hematocrit (%)	40±5
**Echocardiographic and Electrocardiographic Variables**	
Resting Heart Rate (bpm)	66±12
LV End-Diastolic Volume Index (ml/m^2^)	86±29
LV End-Systolic Volume Index (ml/m^2^)	63±25
Mitral Regurgitation Severity (0 to 4)	1.6±1.1
Left Atrial Volume Index (ml/m^2^)	42±16
LV Mass Index (g/m^2^)	151±47

**Table 2.** Differences in HED and NH_3_ Parameter AUC values for predicting SCA

Defect Score (AUC)	Difference in AUC					
	HED Uptake	HED DV	NH3 Uptake	NH3 MBF	Uptake Mismatch	Parametric Mismatch
HED Uptake (0.669)	0.0	+0.015	−0.067	−0.170*	+0.026	+0.059
HED DV (0.684)	-	0.0	−0.082	−0.185*	+0.011	+0.044
NH3 Uptake (0.602)	-	-	0.0	−0.103*	+0.093	+0.125
NH3 MBF (0.499)	-	-	-	0.0	+0.196*	+0.229*
Uptake Mismatch (0.695)	-	-	-	-	0.0	+0.032
Parametric Mismatch (0.727)	-	-	-	-	-	0.0

* P<0.05

**Table 3.** Univariate Cox Proportional Hazards Analysis of HED and NH_3_ defect scores (%LV) for SCA risk

Parameter	Hazard Ratio	Beta Coefficient	Standard Error	P-value
HED Uptake Defect	1.054	0.05220	0.01743	0.003
HED DV Defect	1.061	0.05882	0.01828	0.001
NH_3_ Uptake Defect	1.025	0.02442	0.01816	0.179
NH_3_ MBF Defect	1.000	−0.00011	0.01460	0.994
NH_3_-HED Uptake Mismatch	1.090	0.08617	0.02700	0.001
NH_3_-HED Parametric Mismatch	1.085	0.08187	0.02074	<0.001

## List of Abbreviations

1TCM: One-tissue compartment model
CCS: Canadian Cardiovascular Society
DV: Distribution volume
HED: [^11^C]*meta*-hydroxyephedrine
ICD: Implantable cardioverter-defibrillator
NET-1: Norepinephrine reuptake transporter-1
NH_3_: [^13^N]ammonia
NYHA: New York Heart Association
LV: Left ventricle
LVEF: Left ventricular ejection fraction
MBF: Myocardial blood flow
PAREPET: Prediction of ARrhythmic Events with Positron Emission Tomography
PET: Positron Emission Tomography
SCA: Sudden cardiac arrest
RPC: Coefficient-of-repeatability
