# Supplementary material for: Does Quantification of [11C]meta-hydroxyephedrine and [13N]ammonia Kinetics Improve Risk Stratification in Ischemic Cardiomyopathy
Source: J Nucl Cardiol. Author manuscript; Available in PMC 2023 Apr 1. (PMC8807773; doi:10.1007/s12350-021-02732-5)
Supplement: 1752083_Sup_tab-1 [file NIHMS1752083-supplement-1752083_Sup_tab-1.docx]

**Supplementary Table 1.** Multivariate Cox Proportional Hazards Analysis of HED and NH_3_ defect scores (%LV) for SCA risk

| Parameter/Interaction | Hazard Ratio | Beta Coefficient | Standard Error | P-value |
| --- | --- | --- | --- | --- |
| HED Uptake Defect | 1.061 | 0.05949 | 0.02104 | 0.005 |
| NH_3_ Uptake Defect | 0.986 | −0.01373 | 0.02295 | 0.550 |
|  |  |  |  |  |
| HED Uptake Defect | 1.144 | 0.13452 | 0.05563 | 0.016 |
| NH_3_ Uptake Defect | 1.107 | 0.10156 | 0.08090 | 0.209 |
| NH_3_ Uptake Defect × HED Uptake Defect | 0.996 | −0.00345 | 0.00232 | 0.136 |
|  |  |  |  |  |
| HED DV Defect | 1.082 | 0.07860 | 0.02104 | <0.001 |
| NH_3_ MBF Defect | 0.967 | −0.03365 | 0.01799 | 0.0613 |
|  |  |  |  |  |
| HED DV Defect | 1.128 | 0.12026 | 0.05165 | 0.019 |
| NH_3_ MBF Defect | 1.034 | 0.03371 | 0.07596 | 0.657 |
| NH_3_ MBF Defect × HED DV Defect | 0.998 | −0.00151 | 0.00168 | 0.367 |


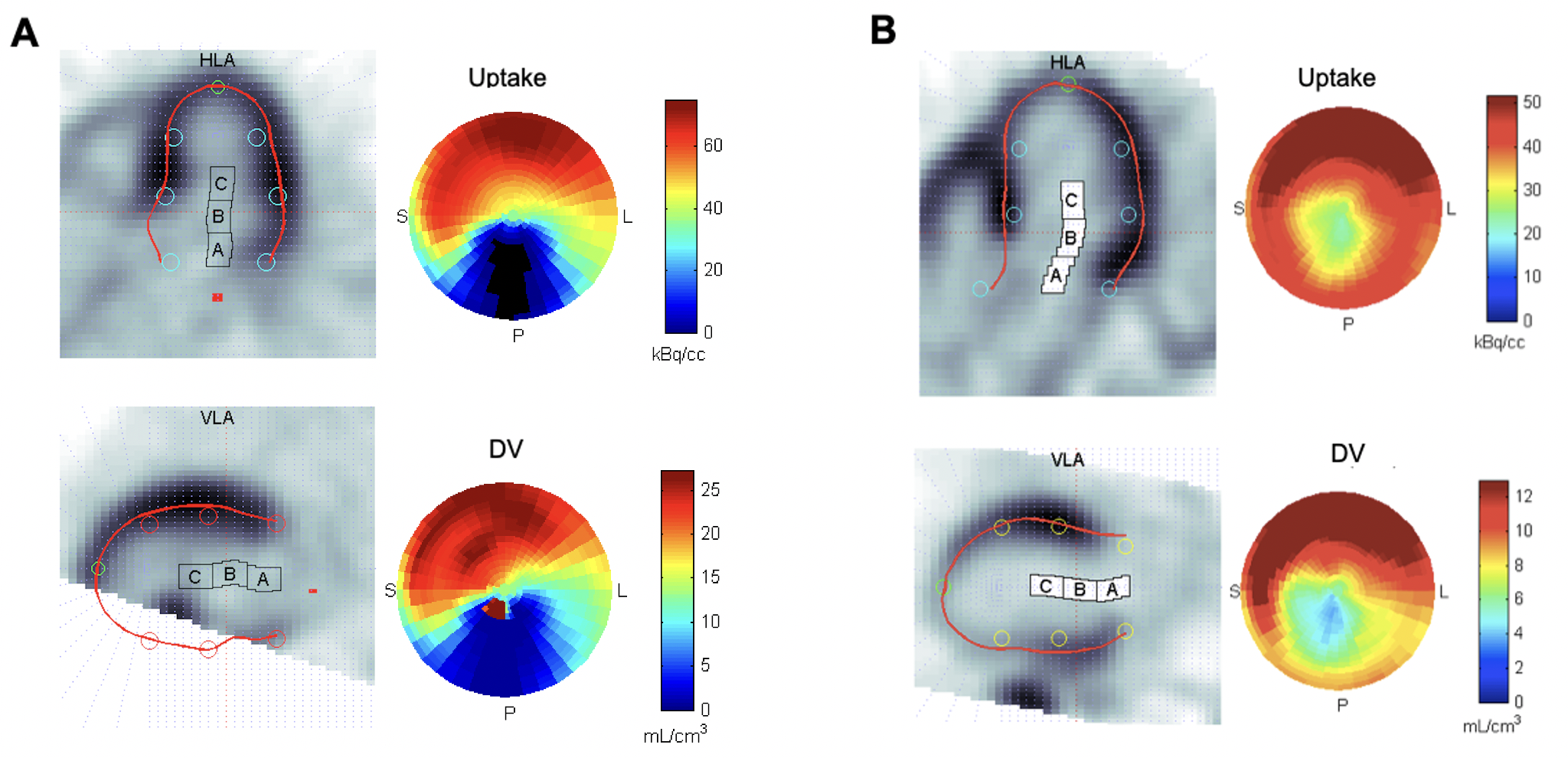


**Supplementary Figure 1**. Example HED polar maps from excluded study participant due to truncated inferior wall (A) compared to included participant (B).


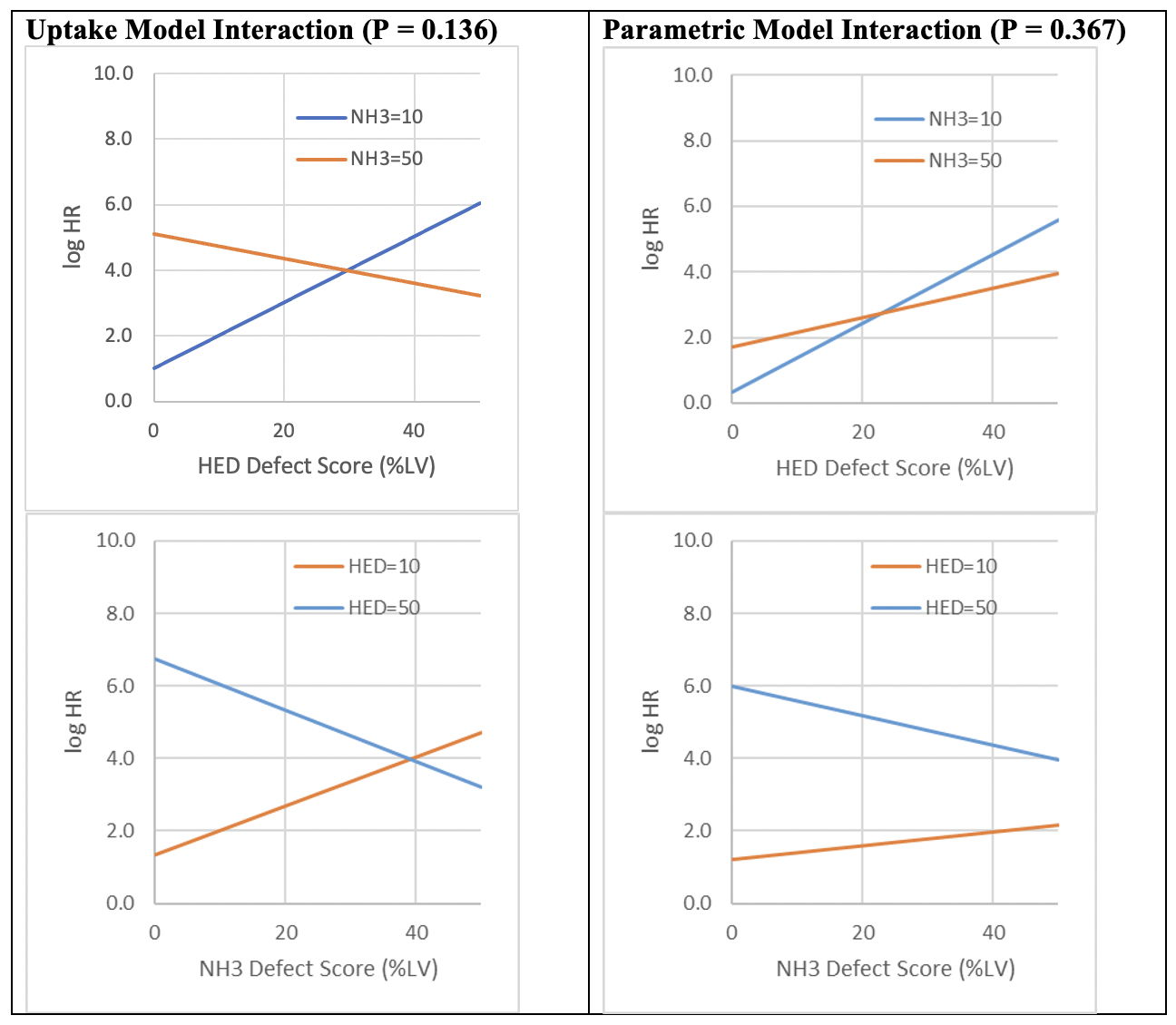
**Supplementary Figure 2**. Cox Proportional Hazards Model Interactions of HED and NH_3_ Defects
