# Supplementary material for: Does Quantification of [11C]meta-hydroxyephedrine and [13N]ammonia Kinetics Improve Risk Stratification in Ischemic Cardiomyopathy
Source: J Nucl Cardiol. Author manuscript; Available in PMC 2023 Apr 1. (PMC8807773; doi:10.1007/s12350-021-02732-5)
Supplement: 1752083_Sup_file-1 [file NIHMS1752083-supplement-1752083_Sup_file-1.pptx]

## Slide 1
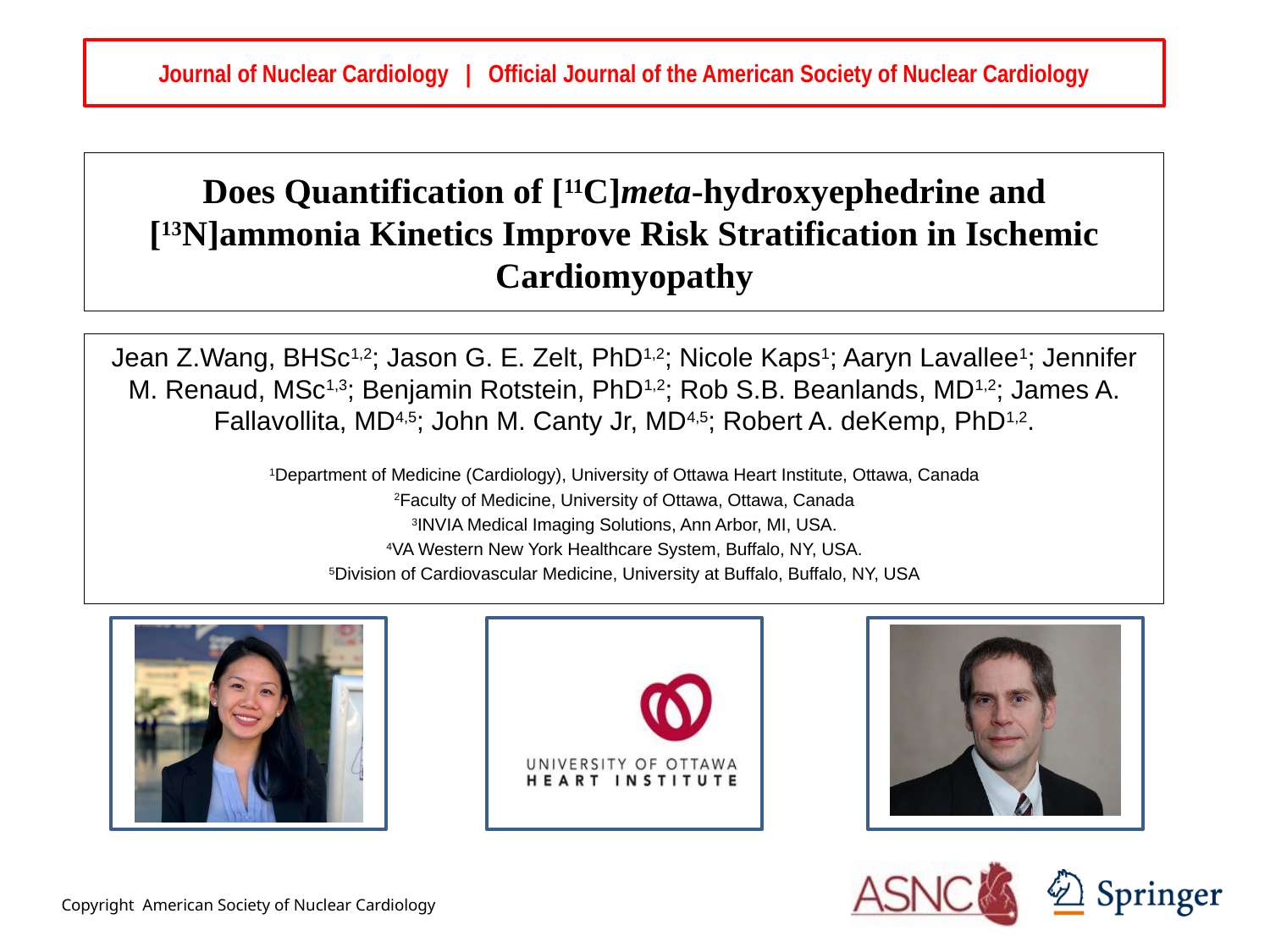

Journal of Nuclear Cardiology | Official Journal of the American Society of Nuclear Cardiology
# Does Quantification of [11C]meta‑hydroxyephedrine and [13N]ammonia Kinetics Improve Risk Stratification in Ischemic Cardiomyopathy
Jean Z.Wang, BHSc1,2; Jason G. E. Zelt, PhD1,2; Nicole Kaps1; Aaryn Lavallee1; Jennifer M. Renaud, MSc1,3; Benjamin Rotstein, PhD1,2; Rob S.B. Beanlands, MD1,2; James A. Fallavollita, MD4,5; John M. Canty Jr, MD4,5; Robert A. deKemp, PhD1,2.
1Department of Medicine (Cardiology), University of Ottawa Heart Institute, Ottawa, Canada
2Faculty of Medicine, University of Ottawa, Ottawa, Canada
3INVIA Medical Imaging Solutions, Ann Arbor, MI, USA.
4VA Western New York Healthcare System, Buffalo, NY, USA.
5Division of Cardiovascular Medicine, University at Buffalo, Buffalo, NY, USA
Copyright American Society of Nuclear Cardiology

## Slide 2
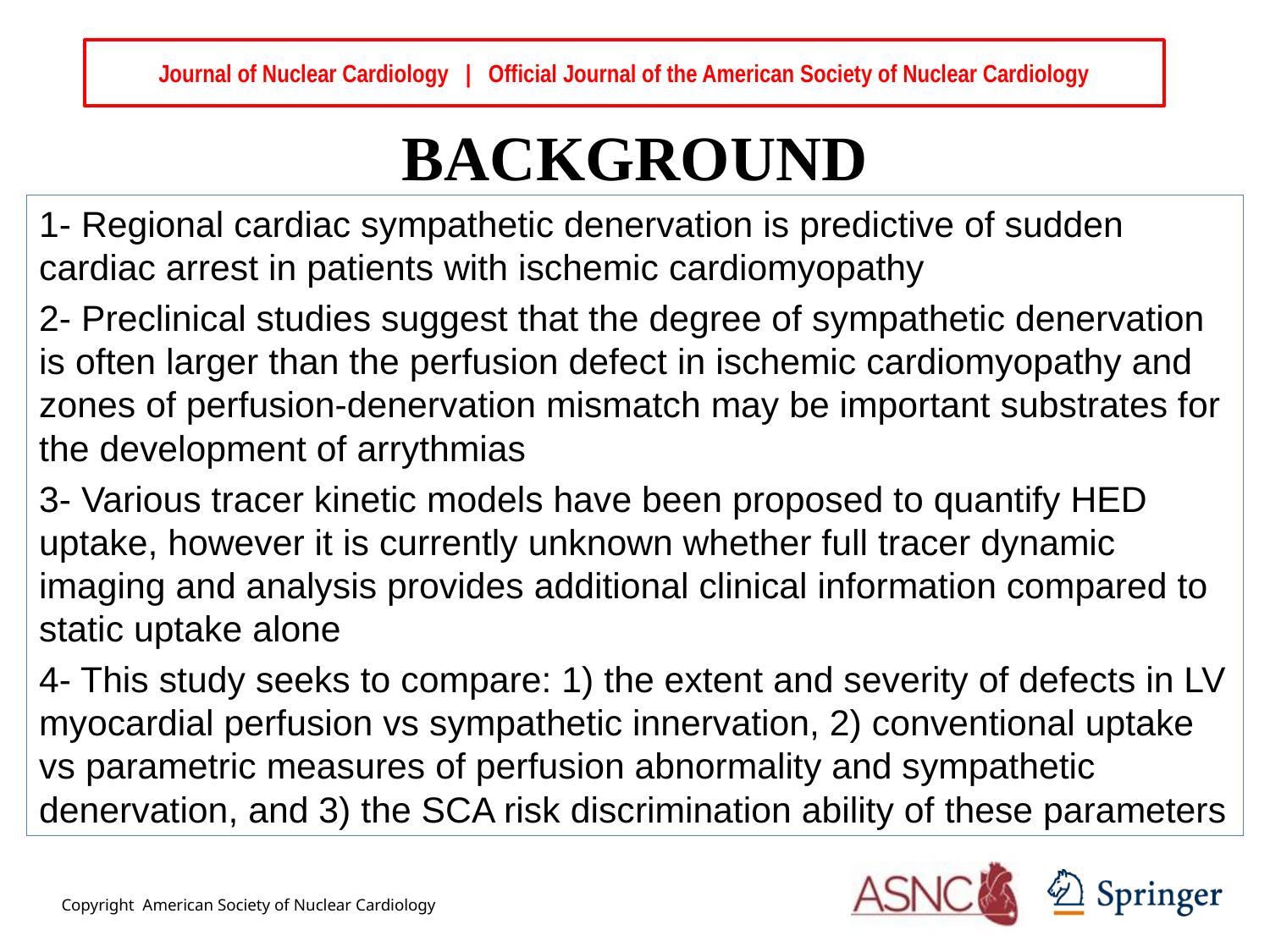

Journal of Nuclear Cardiology | Official Journal of the American Society of Nuclear Cardiology
# BACKGROUND
1- Regional cardiac sympathetic denervation is predictive of sudden cardiac arrest in patients with ischemic cardiomyopathy
2- Preclinical studies suggest that the degree of sympathetic denervation is often larger than the perfusion defect in ischemic cardiomyopathy and zones of perfusion-denervation mismatch may be important substrates for the development of arrythmias
3- Various tracer kinetic models have been proposed to quantify HED uptake, however it is currently unknown whether full tracer dynamic imaging and analysis provides additional clinical information compared to static uptake alone
4- This study seeks to compare: 1) the extent and severity of defects in LV myocardial perfusion vs sympathetic innervation, 2) conventional uptake vs parametric measures of perfusion abnormality and sympathetic denervation, and 3) the SCA risk discrimination ability of these parameters
Copyright American Society of Nuclear Cardiology

## Slide 3
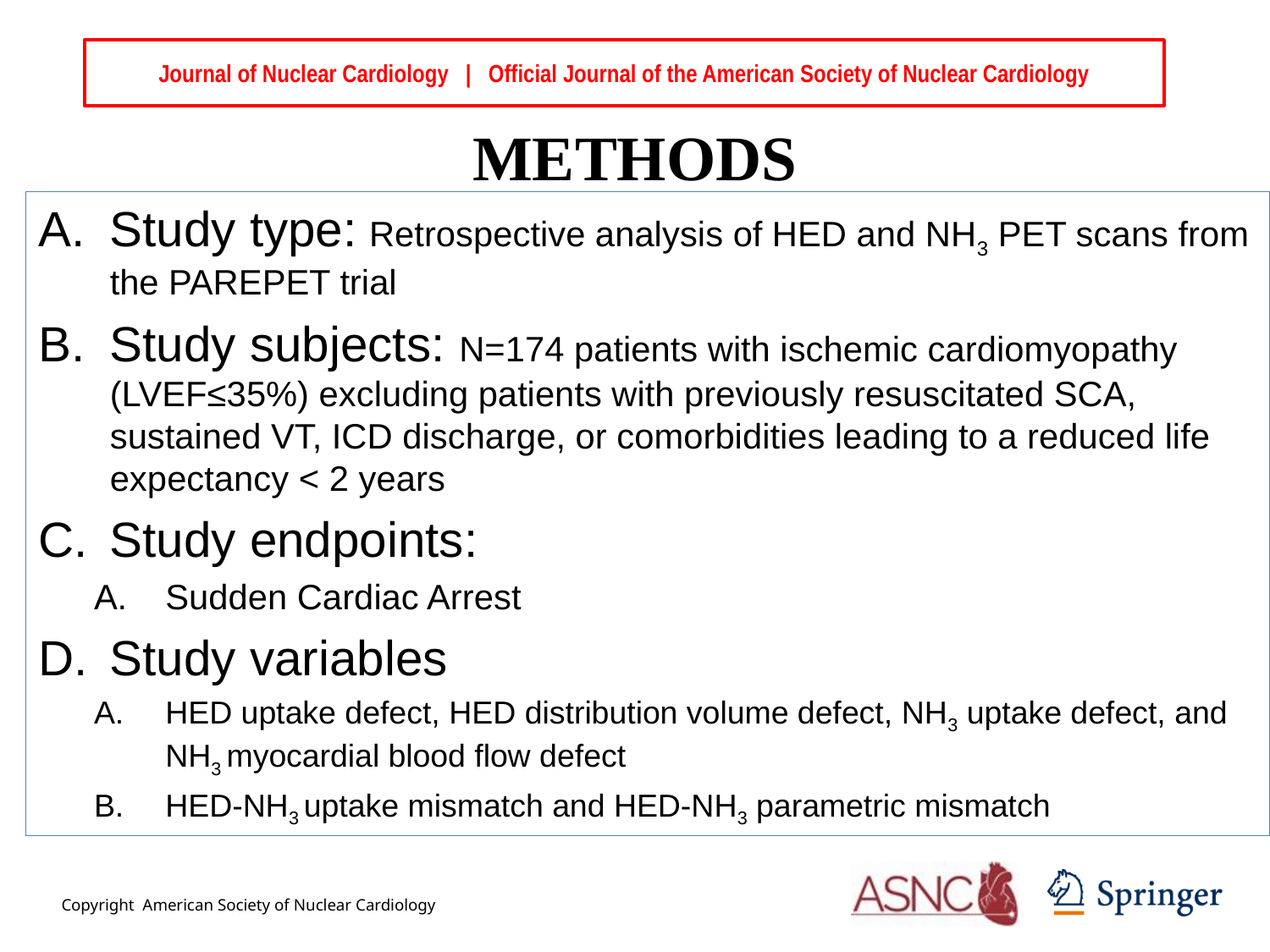

Journal of Nuclear Cardiology | Official Journal of the American Society of Nuclear Cardiology
# METHODS
Study type: Retrospective analysis of HED and NH3 PET scans from the PAREPET trial
Study subjects: N=174 patients with ischemic cardiomyopathy (LVEF≤35%) excluding patients with previously resuscitated SCA, sustained VT, ICD discharge, or comorbidities leading to a reduced life expectancy < 2 years
Study endpoints:
Sudden Cardiac Arrest
Study variables
HED uptake defect, HED distribution volume defect, NH3 uptake defect, and NH3 myocardial blood flow defect
HED-NH3 uptake mismatch and HED-NH3 parametric mismatch
Copyright American Society of Nuclear Cardiology

## Slide 4
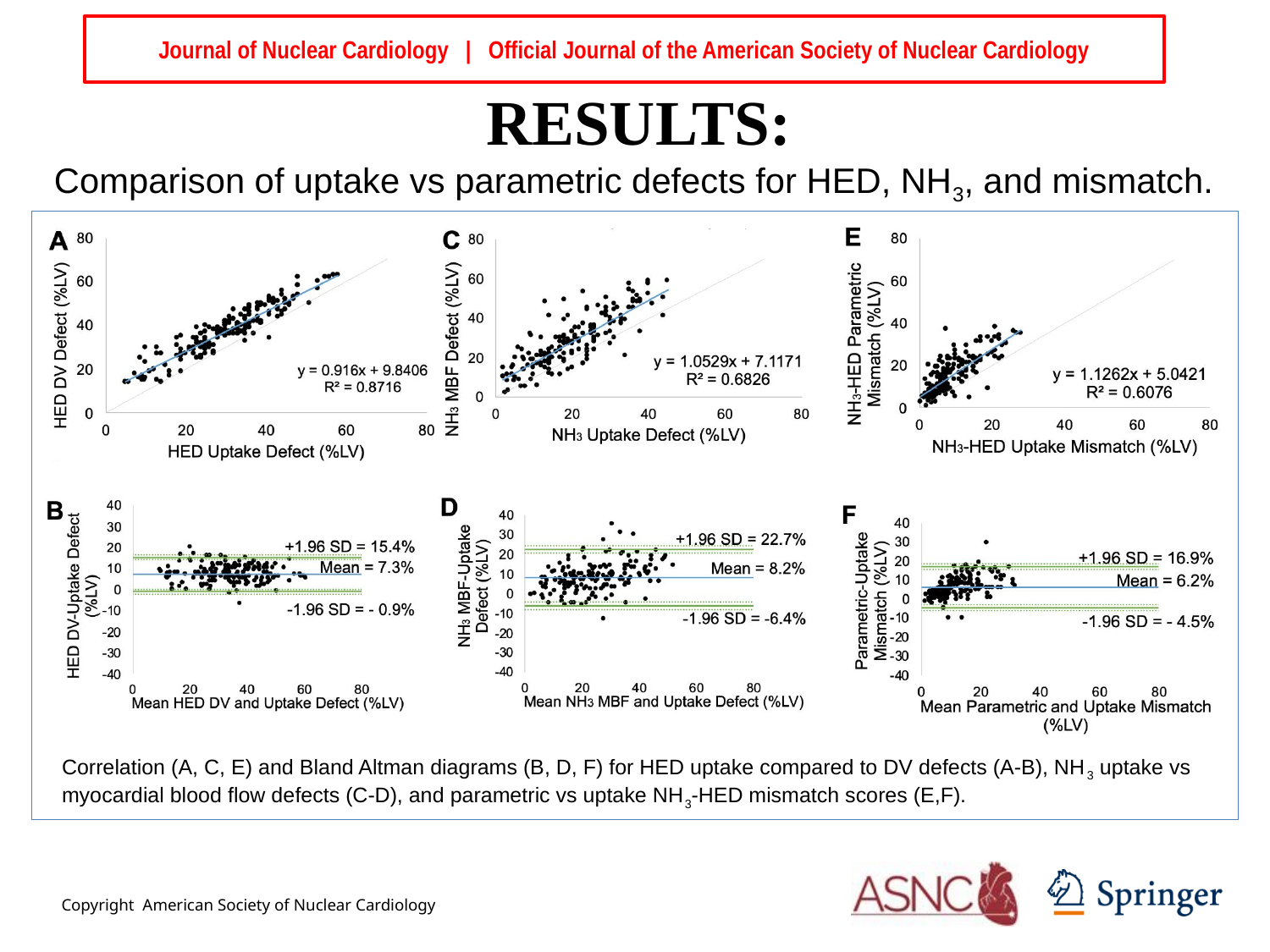

Journal of Nuclear Cardiology | Official Journal of the American Society of Nuclear Cardiology
# RESULTS: Comparison of uptake vs parametric defects for HED, NH3, and mismatch.
Correlation (A, C, E) and Bland Altman diagrams (B, D, F) for HED uptake compared to DV defects (A-B), NH3 uptake vs myocardial blood flow defects (C-D), and parametric vs uptake NH3-HED mismatch scores (E,F).
Copyright American Society of Nuclear Cardiology

## Slide 5
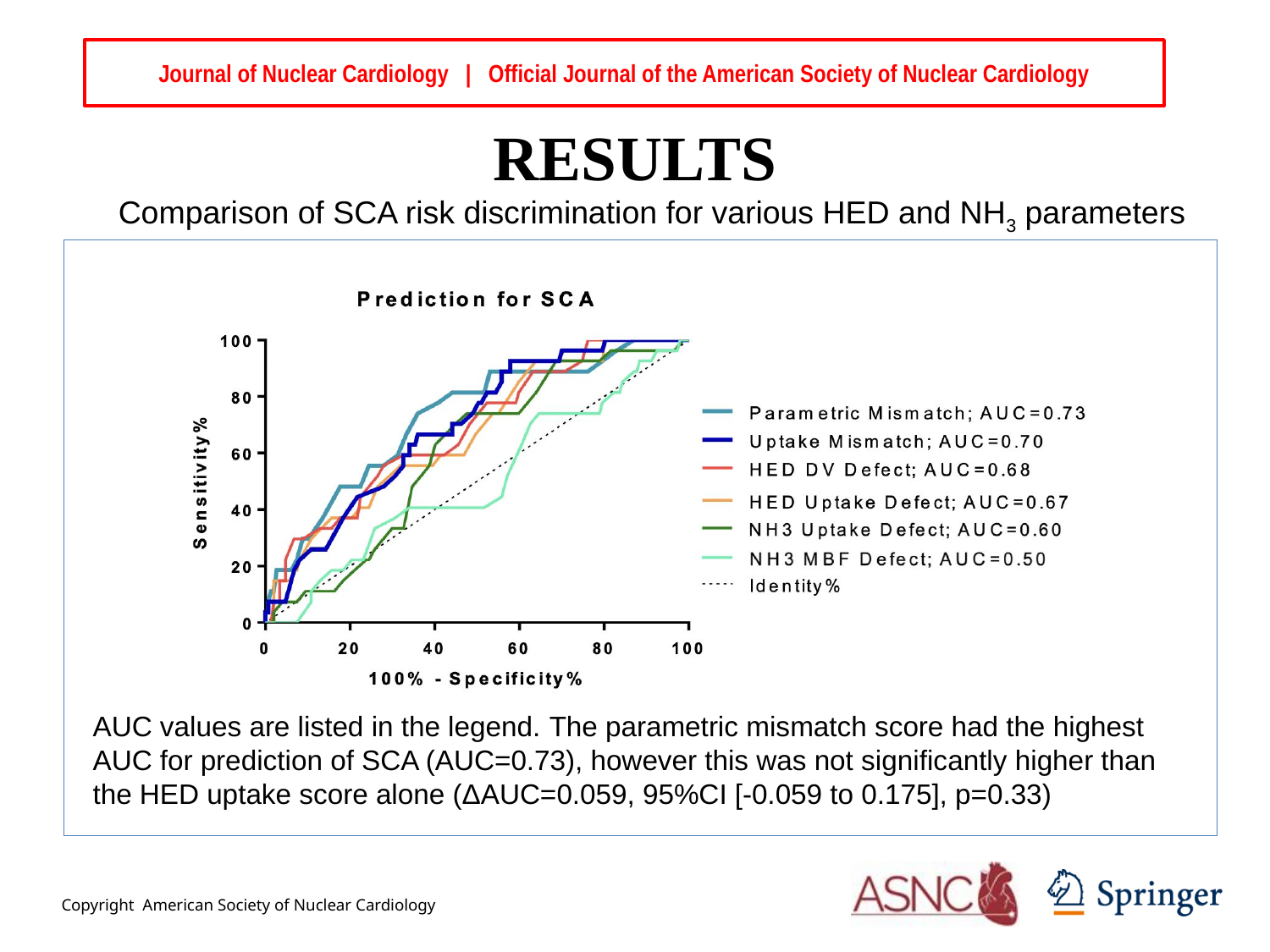

Journal of Nuclear Cardiology | Official Journal of the American Society of Nuclear Cardiology
# RESULTS
Comparison of SCA risk discrimination for various HED and NH3 parameters
AUC values are listed in the legend. The parametric mismatch score had the highest AUC for prediction of SCA (AUC=0.73), however this was not significantly higher than the HED uptake score alone (ΔAUC=0.059, 95%CI [-0.059 to 0.175], p=0.33)
Copyright American Society of Nuclear Cardiology

## Slide 6
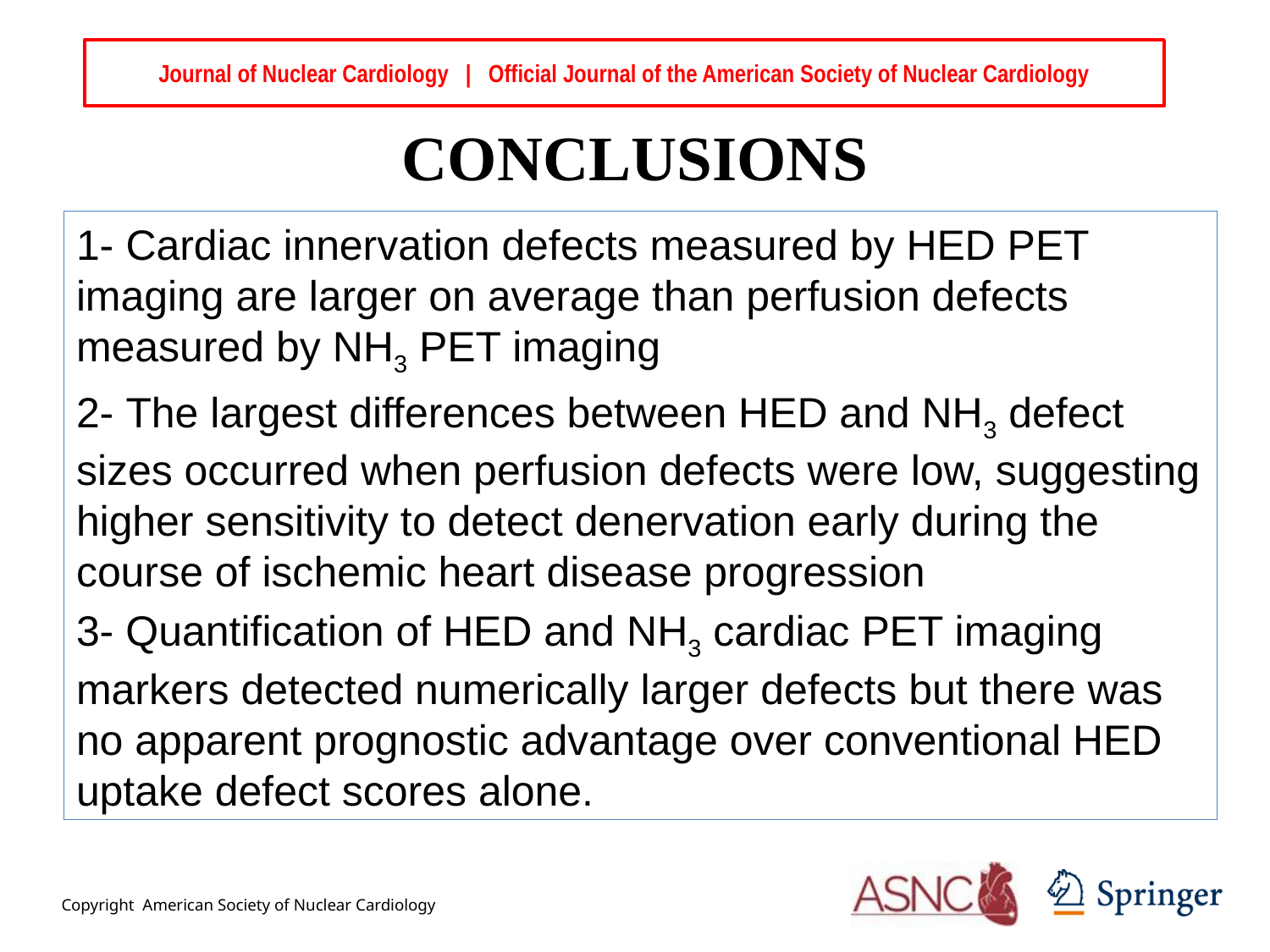

Journal of Nuclear Cardiology | Official Journal of the American Society of Nuclear Cardiology
# CONCLUSIONS
1- Cardiac innervation defects measured by HED PET imaging are larger on average than perfusion defects measured by NH3 PET imaging
2- The largest differences between HED and NH3 defect sizes occurred when perfusion defects were low, suggesting higher sensitivity to detect denervation early during the course of ischemic heart disease progression
3- Quantification of HED and NH3 cardiac PET imaging markers detected numerically larger defects but there was no apparent prognostic advantage over conventional HED uptake defect scores alone.
Copyright American Society of Nuclear Cardiology
