# Supplementary material for: Does Quantification of [11C]meta-hydroxyephedrine and [13N]ammonia Kinetics Improve Risk Stratification in Ischemic Cardiomyopathy
Source: J Nucl Cardiol. Author manuscript; Available in PMC 2023 Apr 1. (PMC8807773; doi:10.1007/s12350-021-02732-5)
Supplement: 1752083_Sup_file-2 [file NIHMS1752083-supplement-1752083_Sup_file-2.docx]

Twitter summary:

Does quantification of cardiac sympathetic denervation and myocardial perfusion improve risk stratification in ischemic cardiomyopathy?

Twitter handles: @HeartInstitute, @UOHI, @DekempRob, @JeanZWang

Hashtags: #cardiacPET
